# Bilateral Temporal Triangular Alopecia Associated with Phakomatosis Pigmentovascularis Type IV Successfully Treated with Follicular Unit Transplantation

**DOI:** 10.1155/2011/129541

**Published:** 2011-09-06

**Authors:** Robin Unger, Mohammed A. Alsufyani

**Affiliations:** ^1^Department of Dermatology, The Mount Sinai School of Medicine, 710 Park Avenue, New York, NY 10021, USA; ^2^Department of Dermatology, Riyadh Military Hospital, Riyadh 11159, Saudi Arabia

## Abstract

Temporal triangular alopecia (TTA), also known as congenital triangular alopecia, is a nonscarring, noninflammatroy, circumscribed form of alopecia. TTA has been associated with several disorders, such as Phakomatosis Pigmentovascularis. Hair restoration surgery using follicular unit transplantation has been a successful treatment modality for TTA. Herein we report such a success that was sustained for over six years.

## 1. Introduction

Temporal triangular alopecia (TTA), also known as congenital triangular alopecia, is a nonscarring, noninflammatory, circumscribed form of alopecia. The alopecic lesion is usually asymptomatic and present at birth or during the first nine years of life. Lesions are stable and mostly presents with roughly triangular, oval, or lancet-shaped patches in the frontotemporal region that are characterized by normal hair density of vellous hair and normal epidermis. The condition mostly occurs unilaterally, but bilateral cases (13.5–20%) can occur [1, 2].

TTA has been associated with several disorders [1], one of which is Phakomatosis Pigmentovascularis [1, 3]. PPVs are rare syndromes characterized by the coexistence of pigmentary nevus and a cutaneous vascular malformation [4, 5].

Hair restoration surgery using follicular unit transplantation has been reported to be a successful treatment for temporal triangular alopecia, where followup for 16 and 24 months postoperatively showed maintenance of the cosmetic end result [2, 6].

## 2. Case Report

A 25-year-old Caucasian male presented to our practice in 2003 complaining of an alopecic scalp lesion in both his temples, which was present since his childhood. The lesions did not increase in size nor did it produce any symptoms.

**Figure 1:**
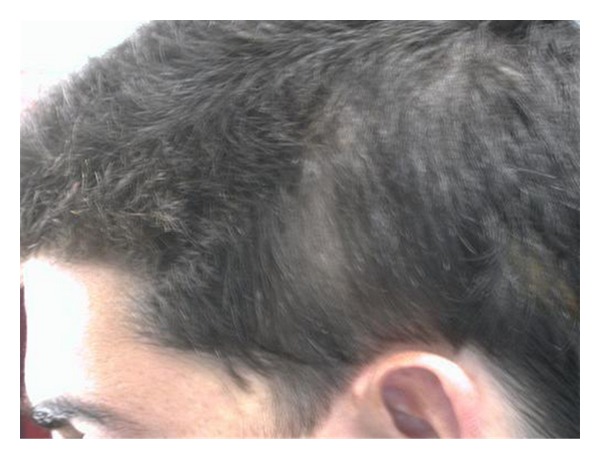
TTA, L. temple.

**Figure 2:**
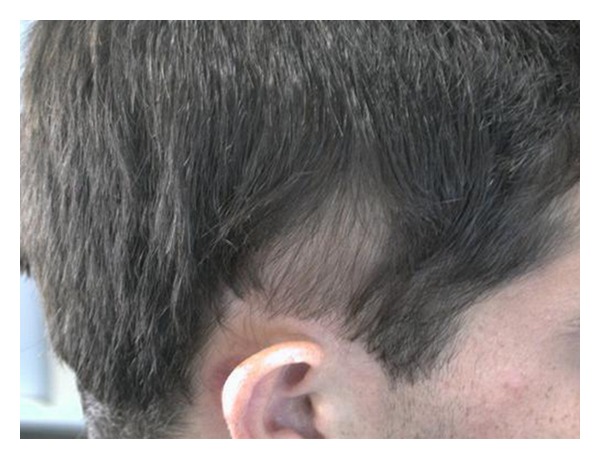
TTA, Right temple.

**Figure 3:**
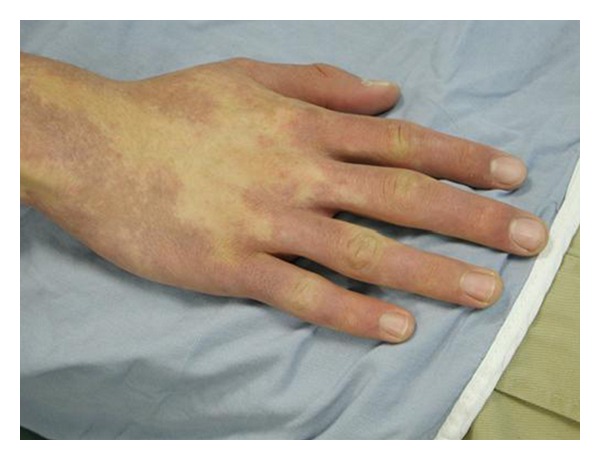
Capillary malformation, Rt. Hand.

**Figure 4:**
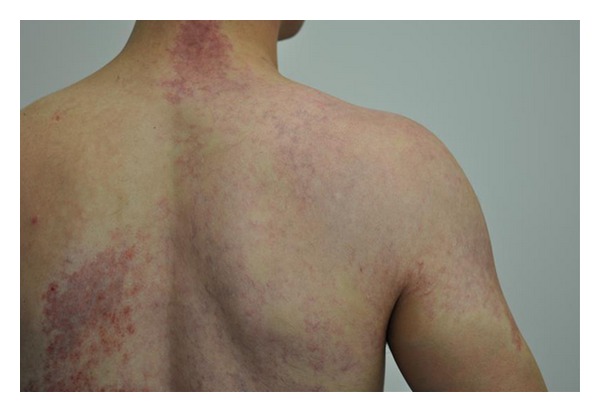
Capillary malformation, back.

**Figure 5:**
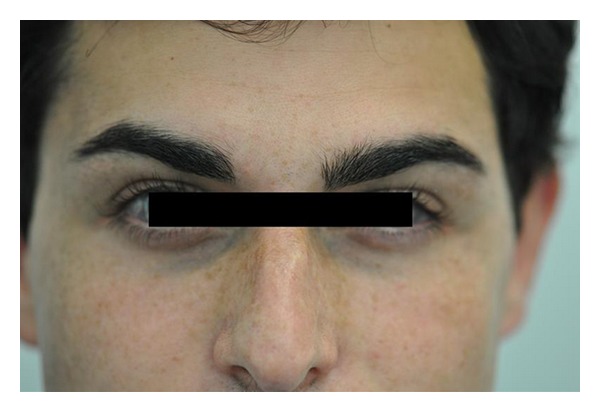
Nevus of Ota.

**Figure 6:**
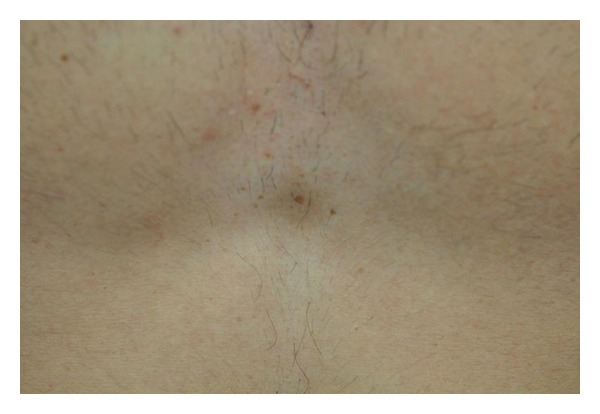
Nevus spilus, chest.

Scalp examination revealed an oval, slightly lancet-shaped hypotrichotic patches measuring approximately 8 × 5 cm and 8 × 4 cm on the left and right temples, respectively, located supra-aurically (Figures 1 and 2). There were no other signs such as erythema, scaling, or atrophy. A 4 mm punch biopsy was taken from the right-sided patch that showed a sparse superficial perivascular and perifollicular infiltrate of lymphocytes. The hair follicles appeared thinner and shorter as well as lipoatrophy. The clinical and histopathological findings were consistent with TTA. The remainder of his cutaneous examination showed the presence of multiple capillary malformations, mostly involving the right upper limb, chest, abdomen, and the back (Figures 3 and 4), for which the patient is receiving laser therapy. In addition, he also had a bilateral nevus of Ota (Figure 5), and a nevus spilus located on the chest (Figure 6). He did not have any other remarkable cutaneous or systemic findings. The constellation of his cutaneous findings (capillary malformation, nevus of Ota, and nevus spilus) led to the clinical diagnosis of Phakomatosis Pigmentovascularis type IVa.

**Figure 7:**
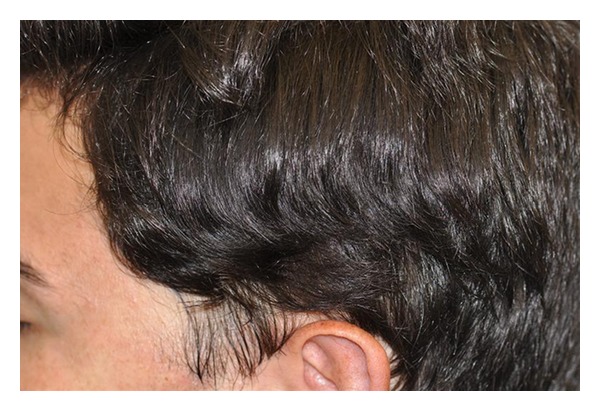
Left side after hair transplant, over six years.

**Figure 8:**
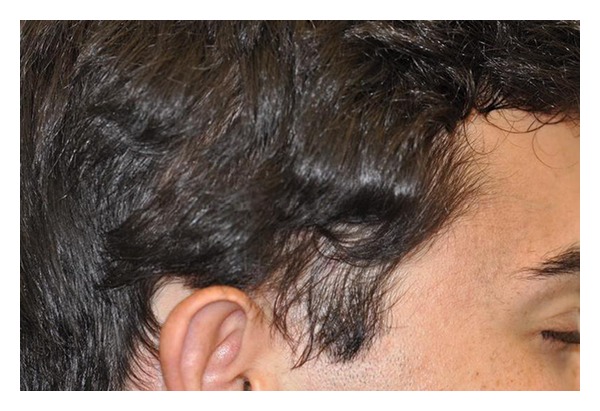
Right side after hair transplant.

**Figure 9:**
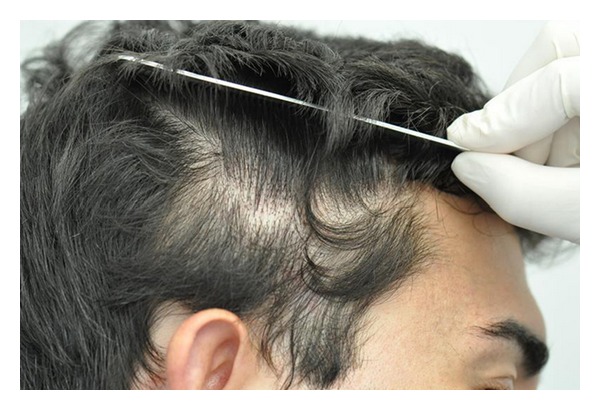
Right side with hair reflected.

Hair restoration surgery with follicular unit transplantation was offered to the patient as a possible treatment which he accepted. The preoperative workout was unremarkable. Patient underwent two sessions one year apart where a 0.7 cm × 5 cm strip was harvested from the occipital donor region, the right half on the first session and the left half on the second session. In total, 1449 follicular units were obtained from both surgeries using stereomicroscopic dissection. The grafts were placed in the TTA patches with a final density of approximately 35 FU/cm^2^. Patient had no postoperative complications after either procedures. Sutures from the donor area were removed seven and ten days after the first and second sessions, respectively. Figures 7, 8 and 9 demonstrate the results after six years after the last surgery. Patient was satisfied with results and did not require any further treatment.

Review of the literature shows, including our case, that hair restoration surgery using follicular unit transplantation is an effective and successful treatment modality for temporal triangular alopecia. In our case report, there was, as well, a consistent result over a six-year followup. We recommend that hair restoration surgery should be offered as a first-line treatment option for this particular type of alopecia.
